# Hypotensive and Endothelium-Dependent Vasorelaxant Effects of Grayblue Spicebush Ethanol Extract in Rats

**DOI:** 10.3390/foods12234282

**Published:** 2023-11-27

**Authors:** Sujin Shin, Junkyu Park, Ho-Young Choi, Kyungjin Lee

**Affiliations:** 1 Department of Korean Medicine, Graduate School, Kyung Hee University, Seoul 02447, Republic of Korea; sjshin04@khu.ac.kr; 2 Department of Science in Korean Medicine, Graduate School, Kyung Hee University, Seoul 02447, Republic of Korea; ojeoksan@khu.ac.kr; 3 Department of Herbal Pharmacology, College of Korean Medicine, Kyung Hee University, Seoul 02447, Republic of Korea; hychoi@khu.ac.kr

**Keywords:** grayblue spicebush, *Lindera glauca*, hypertension, blood pressure, spontaneously hypertensive rat, vasorelaxant, endothelial cells, NO/cGMP pathway, angiotensin II

## Abstract

Hypertension is one of the most common chronic diseases, and its prevalence is increasing worldwide. *Lindera glauca* (Siebold & Zucc.) Blume, known as grayblue spicebush (GS), has been used as food and for medicinal purposes; however, studies about its hypotensive or vasorelaxant effects are lacking. Therefore, the hypotensive effect of an ethanolic extract of the GS branch (GSE) was investigated in 15-week-old spontaneously hypertensive rats (SHRs) using the tail cuff method. The GSE administration group (1000 mg/kg SHR body weight) showed a decrease in their systolic and diastolic blood pressure measured 4 h after its administration. In addition, we investigated its vasorelaxant effect using the thoracic aorta dissected from Sprague-Dawley rats. The GSE (0.5, 1, 2, 5, 10, and 20 μg/mL) showed an endothelium-dependent vasorelaxant effect, and its mechanisms were found to be relevant to the inward rectifier, voltage-dependent, and non-selective K^+^ channels. Moreover, the GSE (20 μg/mL) showed an inhibitory effect on aortic rings constricted with angiotensin II. Considering its hypotensive and vasorelaxant effects, GSE has potential as a functional food to help treat and prevent high blood pressure. However, further studies on the identification of the active components of GSE and safety evaluations of its use are needed.

## 1. Introduction

Hypertension is one of the most common chronic diseases, and its prevalence increased worldwide between 1990 and 2015 [1]. It is an important risk factor for cardiovascular diseases, and calcium channel blockers, angiotensin-converting enzyme inhibitors, or angiotensin receptor blockers are used to treat hypertension [2]. Because of its increasing prevalence and risks, the standard for high blood pressure has been recently lowered to a systolic blood pressure (SBP)/diastolic blood pressure (DBP) higher than 130/80 [3]. However, approximately half of patients with hypertension do not comply with their prescribed blood pressure medication [4]. In addition, 60% of patients experience moderate to severe side effects, such as headaches, dizziness, chest pain, coughing, insomnia, and constipation [5]. Therefore, studies to identify alternatives to the drugs currently used to treat this disease are ongoing [6,7,8].

As the risk factors and prevalence of age-related chronic diseases increase, natural foods have attracted attention because of their possible therapeutic benefits and their safety [9]. Natural foods, including vegetables, fruits, and grains, contain phytochemicals or secondary metabolites that may have therapeutic effects against high blood pressure [10]. For example, angiotensin-converting enzyme inhibitory peptides found in food such as milk, peas, soybeans, and tea have been proposed as alternatives to blood pressure medications [11,12,13]. In addition, the polyphenols found in food enhance cardiovascular health by promoting endothelial function, preventing platelet aggregation, and decreasing inflammation [10,14].

The genus *Lindera*, belonging to the Lauraceae family, is widely distributed in Asia and America [15]. Many *Lindera* plants have been used in tea, seasonings, and fragrances [15,16]; more importantly, they are widely used in traditional medicine. *Lindera glauca* (Siebold & Zucc.) Blume, known as grayblue spicebush (GS), has also been used for various purposes; its leaves have been used as food and tea, its roots as a treatment for bruises and arthritis, and its fruits as a treatment for abdominal pain and paralysis [17]. Recent studies have revealed the various pharmacological effects of the aerial parts of GS, such as its antibacterial [18], antioxidant [19], anti-tumor [20], and neuroprotective effects [21].

However, studies on the hypotensive or vasorelaxant effects of GS are still lacking. Therefore, to investigate the hypotensive effect of a GS branch ethanol extract (GSE), 15-week-old spontaneously hypertensive rats (SHRs), a common model of essential hypertension, were used in this study [22]. The SHR model was selected because over 90% of high blood pressure corresponds to essential hypertension and because SHRs develop high blood pressure over age, similar to humans. They are pre-hypertensive (SBP 100–120 mmHg) for 6–8 weeks, and then become hypertensive (SBP over 150 mm Hg) over 12–14 weeks [22]. In addition, we investigated its vasorelaxant effect using the thoracic aorta dissected from Sprague-Dawley (SD) rats. By pre-constricting the thoracic aorta with phenylephrine (PE), the effect of the GSE on the vascular tone and its underlying mechanisms were studied.

## 2. Materials and Methods

### 2.1. Plant Identification and Preparation of Plant Extract

GS was collected from Aewol-eup, Jeju-si, Jeju, Republic of Korea in December 2021. Morphological identification was performed by Kang-Hyup Lee (Korea National Arboretum) and Weon-Ki Paik (Daejin University, Pocheon-si, Republic of Korea). A voucher specimen was deposited in the College of Korean Medicine, Kyung Hee University (Republic of Korea). GS branches were dried naturally in a well-ventilated place at room temperature. Powdered GS branch (30 g) was extracted by boiling it at 70 ± 2 °C for 2 h with 300 mL of 50% ethanol. After filtering them twice through Hyundai Micro qualitative filter paper No. 2 (Hyundai Micro Co., Ltd., Seoul, Republic of Korea), the extracts were concentrated and freeze-dried. The extract yield of GSE was 6.4%, and GSE was stored in a −20 °C refrigerator.

### 2.2. Animals

Male SHRs (body weight of 300–320 g, 15 weeks old) were obtained from SLC, Inc. (Shizuoka, Japan), and male SD rats (body weight of 230−250 g, 6–7 weeks old) were obtained from Daehan Biolink (Eumseong-gun, Republic of Korea). Animals were housed in polycarbonate cages (22 ± 2 °C, 12/12 h light/dark cycle, humidity 45–65%) and were given free access to feed and water. All experiments complied with the Animal Welfare Guidelines and were approved by the Animal Experiment Ethics Committee of Kyung Hee University (KHSASP-23-340).

### 2.3. Measurement of Blood Pressure in SHR

The SHRs were given distilled water (control group) or GSE (300 and 1000 mg/kg SHR body weight) dissolved in distilled water using an oral zonde needle. Blood pressure in SHR was measured using the tail cuff method as described previously [23]. Measurements of SBP and DBP were performed before administration and at 1, 2, 4, 8, and 12 h after administration.

### 2.4. Chemicals and Solution Preparation

The composition of Krebs–Henseleit (KH) buffer used in this study was as follows: 118.0 mM sodium chloride, 4.7 mM potassium chloride, 2.5 mM calcium chloride, 1.2 mM magnesium sulfate, 1.2 mM monopotassium phosphate, 25.0 mM sodium bicarbonate, and 11.1 mM D-glucose. All reagents used in KH buffer, barium chloride (BaCl_2_), and urethane were purchased from Daejeong Chemical and Gold (Siheung-si, Republic of Korea). Dimethyl sulfoxide (DMSO) was purchased from Junsei (Tokyo, Japan). Acetylcholine (ACh), angiotensin II (Ang II), PE, ethylene glycol bis(2-aminoethyl ether)-N,N,N′,N′-tetraacetic acid (EGTA), indomethacin, and methylene blue (MB) were purchased from Sigma Aldrich (St. Louis, MO, USA). Further, 4-aminopyridine (4-AP), glibenclamide, N^G^-nitro-L-arginine methyl ester (L-NAME), and tetraethylammonium (TEA) were purchased from Wako Pure Chemical Industries (Osaka, Japan), and 1H-[1,2,4] oxadiazolo [4,3-a]quinoxalin-1-one (ODQ) was purchased from the Tokyo Chemical Industry (Tokyo, Japan). 

### 2.5. Measurement of Vascular Relaxation in Rat Aortic Rings

#### 2.5.1. General Experimental Procedures

Preparation of the rat aortic rings was performed as described previously [23]. Briefly, thoracic aorta was extracted after anesthesia and was cut into segments (length: 2–3 mm), which were suspended in chambers. The chambers contained 10 mL KH buffer, were maintained at a temperature of 37 °C, and were supplied with a mixed gas of 95% O_2_ and 5% CO_2_. The aortic rings were stabilized at a tension of 1.0 g for 40–50 min. Measurement of vascular relaxation in rat aortic rings was performed using PowerLab (AD Instrument Co., Bella Vista, Australia).

#### 2.5.2. Vasorelaxant Effect of GSE on the Rat Aortic Rings

GSE was dissolved in DMSO and used for the aorta assay. PE (1 μM) was added to the KH buffer in each chamber after the stabilizing period of 40–50 min to evaluate the vasorelaxant effect of GSE. After blood vessels reached an equilibrium state of maximum contraction due to PE, GSE was added cumulatively (0.5, 1, 2, 5, 10, and 20 μg/mL) to KH buffer in each chamber.

#### 2.5.3. Vasorelaxant Effect of GSE on the Endothelium-Intact or -Denuded Rat Aortic Rings

The endothelium was manually removed through rubbing with cotton to process the endothelium-denuded aortic rings. Endothelial integrity was confirmed using ACh (10 μM) to induce relaxation of vessels pre-contracted using PE (1 μM). The rings that showed relaxant response >85% were defined as endothelium-intact and those that showed relaxant response <10% were defined as endothelium-denuded. After relaxation by ACh, the rings were washed and were contracted with PE (1 μM). After the blood vessels reached an equilibrium state of maximum contraction, GSE (0.5, 1, 2, 5, 10, and 20 μg/mL) was added to the KH buffer in each chamber.

#### 2.5.4. Effects of NO Synthase Inhibitor or Cyclooxygenase (COX) Inhibitor Pretreatment on Vasorelaxation Effect of GSE

Thoracic aortic rings were pretreated with the NO synthase inhibitor L-NAME (100 μM) or the COX inhibitor indomethacin (10 μM) for 20 min after stabilization for 40–50 min. Constriction was induced in the rings using PE (1 μM), and GSE was added (0.5, 1, 2, 5, 10, and 20 μg/mL) to KH buffer in each chamber.

#### 2.5.5. Effects of Soluble Guanylate Cyclase (sGC) Inhibitor or Cyclic Guanosine Monophosphate (cGMP) Inhibitor Pretreatment on Vasorelaxation of GSE

Thoracic aortic rings were pretreated with the sGC inhibitor ODQ (10 μM) or the cGMP inhibitor MB (10 μM) for 20 min after the stabilizing period of 40–50 min. Constriction was induced in the rings using PE (1 μM), and GSE was added (0.5, 1, 2, 5, 10, and 20 μg/mL) to the KH buffer in each chamber.

#### 2.5.6. Effects of K^+^ Channel Blocker Pretreatment on Vasorelaxation of GSE 

Aortic rings were pretreated with the inward rectifier K^+^ channel blocker BaCl_2_ (10 μM), the voltage-dependent K^+^ channel blocker 4-AP (1 mM), the non-selective K^+^ channel blocker TEA (1 mM), or the ATP-sensitive K^+^ channel blocker glibenclamide (10 μM) for 20 min after the stabilizing period of 40–50 min. Constriction was induced in the rings using PE (1 μM), and GSE was added (0.5, 1, 2, 5, 10, and 20 μg/mL) to the KH buffer in each chamber.

#### 2.5.7. Inhibitory Effect of GSE on Aortic Rings Contracted with Extracellular Ca^2+^

To investigate whether GSE has an inhibitory effect on extracellular Ca^2+^ influx, the experiments were conducted in Ca^2+^-free KH buffer. After the equilibration period, aortic rings were incubated with GSE (20 μg/mL) for 20 min and PE (1 μM) for another 20 min. CaCl_2_ was added cumulatively (0.1, 0.3, 1, 3, or 10 mM) to KH buffer in each chamber to induce contractions.

#### 2.5.8. Inhibitory Effect of GSE on Aortic Rings Contracted with Ang II

To investigate whether GSE has an inhibitory effect on Ang II, the rings were incubated with GSE (20 μg/mL) for 20 min. Ang II was added cumulatively (10^−9^, 10^−8^, 10^−7^, 10^−6^ M) to the KH buffer in each chamber to induce contractions.

### 2.6. Statistical Analysis

The results were expressed as the mean ± standard error of the mean (SEM). Statistical significance was assessed using multiple unpaired *t*-tests and two-way analysis of variance (ANOVA), followed by Bonferroni’s multiple comparison test. *p*-values lower than 0.05 were considered statistically significant. All analyses were performed using GraphPad Prism 9 (San Diego, CA, USA).

## 3. Results

### 3.1. Hypotensive Effect of GSE

**Figure 1:**
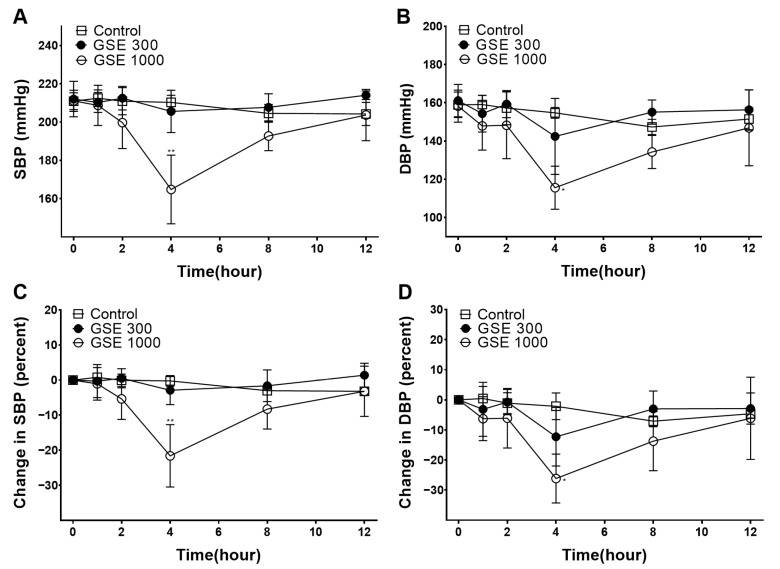
Measured blood pressure values and percentage changes in blood pressure after grayblue spicebush branch ethanol extract (GSE) administration (300 or 1000 mg/kg) in spontaneously hypertensive rats (SHR). (**A**,**B**) Measured systolic blood pressure (SBP) and diastolic blood pressure (DBP) values. (**C**,**D**) percentage changes in SBP and DBP. Values represent mean ± SEM (*n* = 4). * *p* < 0.05, ** *p* < 0.01 vs. control.

To investigate its hypotensive effect, the GSE (300 or 1000 mg/kg SHR body weight) was orally administered to the SHRs, and their blood pressure was measured. In the group administered GSE (1000 mg/kg), their SBP and DBP 4 h after administration were each 164.8 ± 17.9 mmHg and 115.6 ± 11.3 mmHg, respectively, and were significantly lower than those of the group not administered GSE, which was the control group: 210.3 ± 3.7 mmHg and 154.8 ± 2.8 mmHg (Figure 1A,B). In addition, changes in SBP and DBP after the administration of the GSE compared to SBP and DBP before its administration in each SHR were calculated as percentages and compared with those in the control group. The percentage changes in SBP and DBP 4 h after the administration of 1000 mg/kg were −21.6 ± 8.9% and −26.2 ± 8.1% and were also significantly lower than those in the SBP and DBP of the control group: −0.2 ± 1.5% and −2.1 ± 4.5% (Figure 1C,D).

### 3.2. The Vasorelaxant Effect of GSE on the Rat Aortic Rings

**Figure 2:**
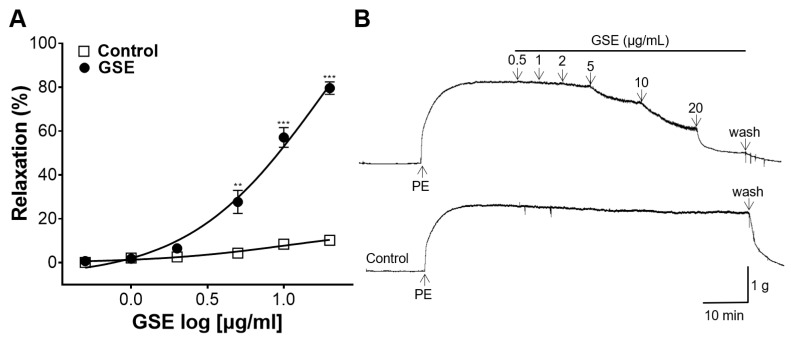
Vasorelaxant effect of grayblue spicebush branch ethanol extract (GSE). (**A**) Concentration–response curves and (**B**) isotonic changes in aortic rings contracted with phenylephrine (PE). Values represent mean ± SEM (*n* = 4–5). ** *p* < 0.01, *** *p* < 0.001 vs. control.

The rings were constricted using PE, and the GSE (0.5, 1, 2, 5, 10, and 20 μg/mL) was added after the blood vessels reached an equilibrium state of maximum contraction. The GSE showed a significant concentration-dependent vascular relaxant effect (Figure 2). 

### 3.3. Vasorelaxant Effect of GSE on the Endothelium-Intact or -Denuded Rat Aortic Rings

**Figure 3:**
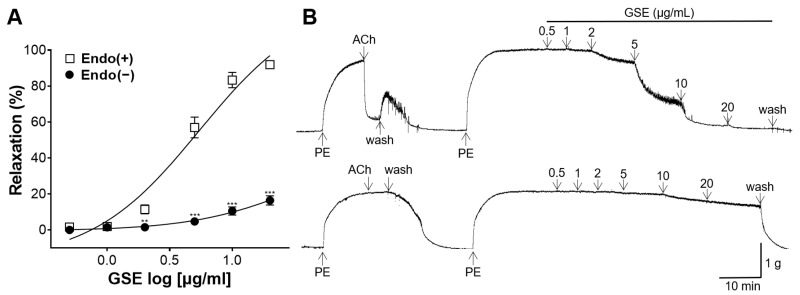
Vasorelaxant effect of grayblue spicebush branch ethanol extract (GSE) on endothelium-intact [Endo(+)] or -denuded [Endo(−)] rat aortic rings. (**A**) Concentration–response curves and (**B**) isotonic changes in aortic rings contracted with phenylephrine (PE). Values are mean ± SEM (*n* = 4). ** *p* < 0.01, *** *p* < 0.001 vs. control.

To confirm endothelium-dependent vasorelaxation, endothelium-intact or -denuded aortic rings were treated with GSE. In the endothelium-intact aortic rings, the GSE (0.5, 1, 2, 5, 10, and 20 μg/mL) showed a dose-dependent vascular relaxant effect, with a maximum effect of 91.90 ± 0.34% at 20 μg/mL (Figure 3). However, in the endothelium-denuded aortic rings, the GSE showed little vascular relaxant effect, with a maximum effect of 16.40 ± 2.56% at 20 μg/mL (Figure 3).

### 3.4. Effects of NO Synthase Inhibitor or COX Inhibitor Pretreatment on Vasorelaxation of GSE

**Figure 4:**
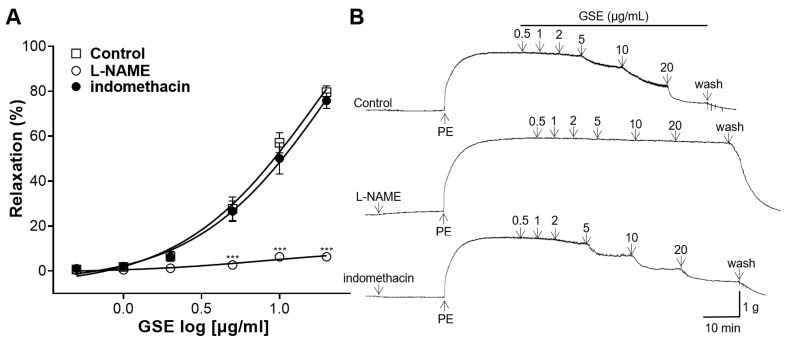
Effects of N^G^-nitro-L-arginine methyl ester (L-NAME) or indomethacin pretreatment on vasorelaxation effect of grayblue spicebush branch ethanol extract (GSE). (**A**) Concentration–response curves and (**B**) isotonic changes in aortic rings contracted with phenylephrine (PE). Values are mean ± SEM (*n* = 4–5). *** *p* < 0.001 vs. control.

The NO synthase inhibitor L-NAME or the COX inhibitor indomethacin were used for the pretreatment of the rat aortic rings. The vasorelaxant effects of GSE (0.5, 1, 2, 5, 10, and 20 μg/mL) on the aortic rings pretreated with inhibitors were compared with those of the controls. In the rings pretreated with L-NAME, the GSE had a significantly reduced vasodilatory effect. However, there was no significant difference between the rings pretreated with indomethacin and the control group (Figure 4).

### 3.5. Effects of the sGC Inhibitor or the cGMP Inhibitor Pretreatment on Vasorelaxation of GSE

**Figure 5:**
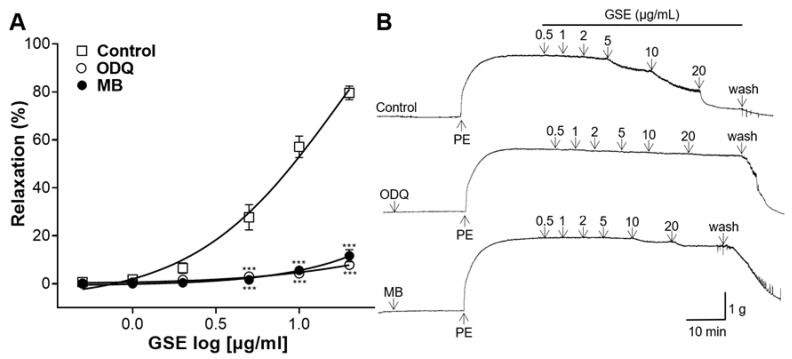
Effects of 1H-[1,2,4] Oxadiazolo [4,3-a]quinoxalin-1-one (ODQ) or methylene blue (MB) pretreatment on vasorelaxation of grayblue spicebush branch ethanol extract (GSE). (**A**) Concentration–response curves and (**B**) isotonic changes in aortic rings contracted with phenylephrine (PE). Values are mean ± SEM (*n* = 4–5). *** *p* < 0.001 vs. control.

The sGC inhibitor ODQ or the cGMP inhibitor MB were used to pretreat the rat aortic rings. The vasorelaxant effect of the GSE (0.5, 1, 2, 5, 10, and 20 μg/mL) on the aortic rings pretreated with inhibitors was compared with that of the control group. In the aortic rings pretreated with the sGC inhibitor or the cGMP inhibitor, the GSE had a significantly reduced vasodilatory effect compared to that of the control (Figure 5).

### 3.6. Effects of K^+^ Channel Blocker Pretreatment on Vasorelaxation of GSE

**Figure 6:**
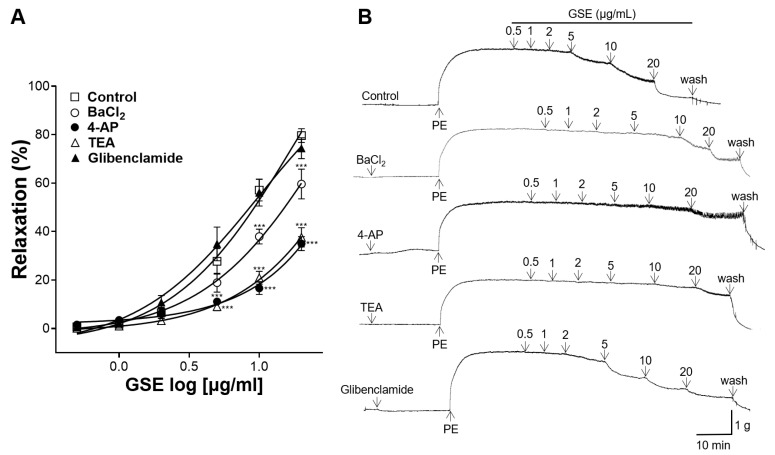
Effects of barium chloride (BaCl_2_), 4-aminopyridine (4-AP), tetraethylammonium (TEA), or glibenclamide pretreatment on vasorelaxation effect of grayblue spicebush branch ethanol extract (GSE). (**A**) Concentration–response curves and (**B**) isotonic changes in aortic rings contracted with phenylephrine (PE). Values are mean ± SEM (*n* = 4–5). *** *p* < 0.001 vs. control.

K^+^ channels play an important role in determining vascular tone and diameter by regulating the membrane potential of vascular smooth muscle cells (VSMCs). Accordingly, thoracic aortic rings were pretreated with blockers of the four main types of K^+^ channels: the inward rectifier K^+^ channel blocker BaCl_2_, the voltage-dependent K^+^ channel blocker 4-AP, the non-selective K^+^ channel blocker TEA, or the ATP-sensitive K^+^ channel blocker glibenclamide. The vasorelaxant effect of the GSE (0.5, 1, 2, 5, 10, and 20 μg/mL) on the aortic rings pretreated with K^+^ channel blockers were compared with that of the control group. In the aortic rings pretreated with BaCl_2_, 4-AP, or TEA, the GSE had significantly reduced vasodilatory effects; however, there was no significant difference between the rings pretreated with glibenclamide and the control group (Figure 6).

### 3.7. Inhibitory Effect of GSE on Rat Aortic Rings Constricted with Extracellular Ca^2+^

**Figure 7:**
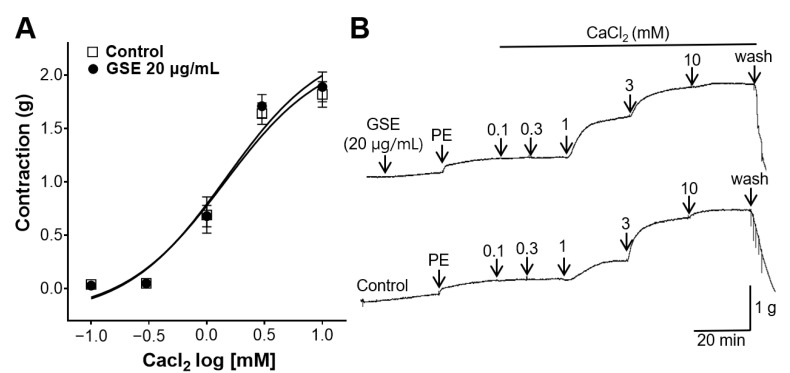
Inhibitory effect of grayblue spicebush branch ethanol extract (GSE) on thoracic aortic rings contracted with extracellular CaCl_2_. (**A**) Inhibitory effect of GSE on CaCl_2_ contraction and (**B**) isotonic changes in aortic rings. Values are mean ± SEM (*n* = 5).

Before inducing constriction, GSE (20 μg/mL) was added to the aortic rings for 20 min. Then, CaCl_2_ was added cumulatively to induce contractions. However, no significant difference was observed between the rings pretreated with the GSE and the control group without a pretreatment (Figure 7).

### 3.8. Inhibitory Effect of GSE on Rat Aortic Rings Constricted with Ang II

**Figure 8:**
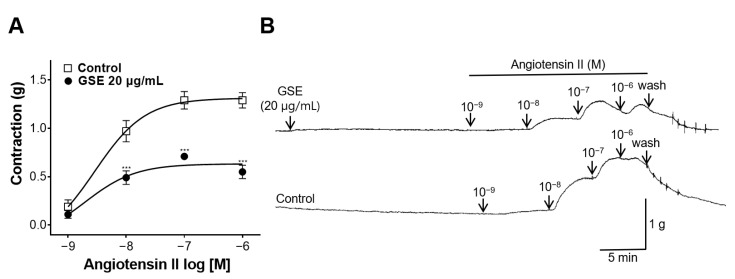
Inhibitory effect of grayblue spicebush branch ethanol extract (GSE) on thoracic aortic rings contracted using angiotensin II (Ang II). (**A**) Inhibitory effect of GSE on Ang II contraction and (**B**) isotonic changes in aortic rings. Values are mean ± SEM (*n* = 5). *** *p* < 0.001 vs. control.

Before inducing constrictions, GSE (20 μg/mL) was added to the aortic rings. Then, Ang II was cumulatively added to induce contractions. The aortic rings pretreated with GSE showed inhibitory effects on vasoconstriction compared with the control group without a pretreatment (Figure 8).

## 4. Discussion

In the present study, the hypotensive and vasorelaxant effects of GSE were investigated. To investigate the hypotensive effect of GSE, 300 or 1000 mg/kg of GSE was orally administered to SHRs. The blood pressure of the SHRs was measured before and 1, 2, 4, 8, and 12 h after administration using the tail cuff method. Consequently, in the group administered GSE (1000 mg/kg), their SBP and DBP measured 4 h after administration decreased significantly compared to those of the control group. Because previous studies have revealed that substances that have a hypotensive effect in SHR are also effective in humans with high blood pressure [22], our results suggest that GSE shows potential as a functional food for hypertension. When the dose administered to rats in our experiments was converted to the human-equivalent dose (for an adult male with a body weight of 60 kg), a dose of approximately 3–10 g of GSE could be applied [24]. However, animal experiments and clinical trials with various doses are needed to determine the optimal dose in humans.

GS has long been used in tea, food, and traditional medicine and is known to have a low toxicity [25]. In a previous in vivo study of GS, an acute toxicity test of the ethyl acetate fraction of the GS branch was performed, and it was found to be non-toxic up to an oral administration dose of 2000 mg/kg [26]. SHRs that were administered GSE (300 or 1000 mg/kg) in our experiment also did not show side effects; however, detailed toxicity studies are required to determine the safe dosage in humans.

Blood vessels mediate circulatory blood flow by regulating vascular tone and lumen diameter through various mechanisms [27]. Vasodilation is involved in reducing systemic vascular resistance and blood pressure, and the dysfunction of vascular relaxation can lead to hypertension [28]. Therefore, the vasorelaxant effect of GSE was investigated using the thoracic aorta dissected from SD rats in this study. According to our experimental results, GSE showed a significant dose-dependent vasorelaxant effect on PE-constricted aortic rings compared to that in control. Vascular endothelial cells play an important role in determining the physiological and pathological status of the cardiovascular system including hypertension [29]. To confirm the endothelium-dependent vasorelaxant effect of GSE, GSE was added to each endothelium-intact or endothelium-denuded aortic ring. In the endothelium-intact aortic rings, the GSE showed a maximum effect of 91.90 ± 0.34% at 20 μg/mL; however, in the endothelium-denuded aortic rings, the GSE showed a smaller vascular relaxant effect of 16.40 ± 2.56% at the same concentration. Therefore, our findings suggested that GSE exerts its vasorelaxant effects primarily via an endothelium-dependent mechanism.

Vascular endothelial cells mediate vasorelaxation by secreting substances including the NO produced by NO synthase and prostacyclin (PGI_2_) produced by COX [30]. NO and PGI_2_ activate sGC and adenyl cyclase, respectively, to induce cGMP and adenosine monophosphate [31]. To determine whether the vasorelaxant effect of GSE is relevant to NO or COX, thoracic aortic rings were pretreated with the NO synthase inhibitor L-NAME or the COX inhibitor indomethacin. In the aortic rings pretreated with L-NAME, the GSE had a significantly reduced vasodilatory effect. However, there was no significant difference between the rings pretreated with indomethacin and those in the control group. Therefore, our results suggest that the vasorelaxant effect of GSE is relevant to NO secretion and is independent of PGI_2_.

sGC and the produced cGMP play a crucial role in vasorelaxation by lowering the Ca^2+^ concentration in VSMCs [30]; therefore, sGCs are an important target for the treatment of the cardiovascular system [32]. To determine whether GSE is relevant to the NO-sGC-cGMP pathway, rings were pretreated with the sGC inhibitor ODQ or the cGMP inhibitor MB. In our study, in the aortic rings pretreated with the sGC or cGMP inhibitor, the GSE had a significantly reduced vasodilatory effect compared to that of the control group. Therefore, our results suggested that GSE acts on the NO-sGC-cGMP pathway.

Regulating the membrane ion channels of VSMCs, for example, by activating K^+^ channels and blocking Ca^2+^ channels, is also a mechanism of vasodilation [33]. To investigate the effect of GSE on K^+^ channels, aortic rings were pretreated with the following K^+^ channel blockers: the inward rectifier K^+^ channel blocker, BaCl_2_; voltage-dependent K^+^ channel blocker, 4-AP; non-selective K^+^ channel blocker, TEA; or ATP-sensitive K^+^ channel blocker, glibenclamide. The pretreatment of the aortic rings with BaCl_2_, 4-AP, or TEA significantly reduced the vasodilatory effects of the GSE. Therefore, our results suggest that GSE acts on inward rectifier, voltage-dependent, and non-selective K^+^ channels. However, no significant differences were observed between the rings pretreated with glibenclamide and those in the control group. Generally, ATP-sensitive K^+^ channels have little effect on the vascular tone [34], and our results showed that ATP-sensitive K^+^ channels have little influence on the vascular relaxation effect of GSE.

The influx of Ca^2+^ through voltage-dependent or receptor-operated Ca^2+^ channels causes blood vessel constriction [35]. To investigate whether GSE is associated with the blockage of Ca^2+^ channels, aortic rings were treated with GSE (20 μg/mL), and CaCl_2_ was added cumulatively to induce constrictions. However, no significant differences were observed between the rings pretreated with the GSE and those in the control group. Therefore, our results indicated that the vasorelaxant effect of the GSE was not relevant in terms of Ca^2+^ channels.

The renin–angiotensin system is associated with blood pressure control and Ang II is the major effector peptide involved therein [36]. Ang II regulates vascular tone by producing an immediate vasoconstrictive effect and is related to endothelial dysfunction, leading to hypertension [37]. To investigate the inhibitory effect of GSE on blood vessels contracted with Ang II, aortic rings were treated with GSE (20 μg/mL), and Ang II was added cumulatively to induce constrictions. The aortic rings treated with the GSE showed inhibitory effects on vasoconstriction, indicating that the GSE interfered with the action of Ang II.

Natural foods have been found to exert vasorelaxant effects through various mechanisms [38,39]. GSE also exhibits a vasorelaxant effect via the activation of the NO/cGMP pathway and K^+^ channels. Studies have shown that endothelium-dependent vasorelaxation may activate K^+^ channels in VSMCs [34]. Therefore, these two GSE mechanisms may interact with each other. Additionally, GSE inhibits the vasoconstrictive action of Ang II. Therefore, GSE is a valuable source of natural foods for the treatment and prevention of hypertension. However, the composition and content of GS were not investigated in this study. Further studies, including the identification of the bioactive components in GSE and toxicity evaluations, are required.

## 5. Conclusions

In conclusion, the administration of 1000 mg/kg of GSE showed a significantly reduced SBP and DBP in SHRs. In addition, GSE showed a vasorelaxant effect via the NO/cGMP pathway and by inward rectifier, voltage-dependent, and non-selective K^+^ channels. Additionally, it showed inhibitory effects on vessels contracted with Ang II. Considering its hypotensive and vasorelaxant effects, GSE shows potential as a functional food to help treat and prevent high blood pressure. However, further research studies are needed to evaluate the safety of its use and for the identification of its constituents and active components.

## Data Availability

The data presented in this study are available from the corresponding author upon request.
